# Remote Monitoring of Cardiac Implantable Electronic Devices in Patients Undergoing Hybrid Comprehensive Telerehabilitation in Comparison to the Usual Care. Subanalysis from Telerehabilitation in Heart Failure Patients (TELEREH-HF) Randomised Clinical Trial

**DOI:** 10.3390/jcm9113729

**Published:** 2020-11-20

**Authors:** Sławomir Pluta, Ewa Piotrowicz, Ryszard Piotrowicz, Ewa Lewicka, Wojciech Zaręba, Monika Kozieł, Ilona Kowalik, Michael J. Pencina, Artur Oręziak, Andrzej Cacko, Dominika Szalewska, Renata Główczyńska, Maciej Banach, Grzegorz Opolski, Piotr Orzechowski, Robert Irzmański, Zbigniew Kalarus

**Affiliations:** 1 Department of Cardiology and Angiology, Silesian Centre for Heart Disease, 41-800 Zabrze, Poland; spluta77@gmail.com (S.P.); kozielmonika@poczta.fm (M.K.); 2 Telecardiology Center, National Institute of Cardiology, 04-628 Warsaw, Poland; porzechowski@ikard.pl; 3 National Institute of Cardiology, 04-628 Warsaw, Poland; rpiotrowicz@ikard.pl (R.P.); ikowalik@ikard.pl (I.K.); 4 Warsaw Academy of Medicine Rehabilitation, 02-091 Warsaw, Poland; 5 Department of Cardiology and Electrotherapy, Medical University of Gdańsk, 80-211 Gdańsk, Poland; elew@gumed.edu.pl; 6 Department of Medicine, University of Rochester Medical Center, Rochester, NY 14642, USA; Wojciech_Zareba@urmc.rochester.edu; 7 Department of Biostatistics and Bioinformatics, Duke University School of Medicine, Durham, NC 27707, USA; michal.pencina@duke.edu; 8 Department of Arrhythmia, National Institute of Cardiology, 04-628 Warsaw, Poland; areziak@ikard.pl; 9 Department of Medical Informatics and Telemedicine, Medical University of Warsaw, 02-091 Warsaw, Poland; andrzej.cacko@gmail.com; 10 Rehabilitation Medicine, Medical University of Gdańsk, 80-211 Gdańsk, Poland; dominika.szalewska@gumed.edu.pl; 11 Department of Cardiology, Medical University of Warsaw, 02-091Warsaw, Poland; reniarenata@gmail.com (R.G.); Grzegorz.Opolski@wum.edu.pl (G.O.); 12 Department of Hypertension, Medical University of Łódź, 92-213 Łódź, Poland; maciej.banach@icloud.com; 13 Department of Internal Medicine and Cardiac Rehabilitation, Medical University of Łódź, 92-213 Łódź, Poland; robert.irzmanski@umed.lodz.pl; 14 Department of Cardiology, Congenital Heart Diseases and Electrotherapy, Division of Medical Sciences in Zabrze, Medical University of Silesia, 41-800 Zabrze, Poland; zbigniewkalarus@kalmet.com.pl

**Keywords:** cardiac implantable electronic devices, hybrid comprehensive telerehabilitation, remote monitoring, heart failure

## Abstract

Background: The impact of cardiac rehabilitation on the number of alerts in patients with remote monitoring (RM) of cardiac implantable electronic devices (CIEDs) is unknown. We compared alerts in RM and outcomes in patients with CIEDs undergoing hybrid comprehensive telerehabilitation (HCTR) versus usual care (UC). Methods: Patients with heart failure (HF) after a hospitalization due to worsening HF within the last 6 months (New York Heart Association (NYHA) class I-III and left ventricular ejection fraction (LVEF) ≤40%) were enrolled in the TELEREH-HF study and randomised 1:1 to HCTR or UC. Patients with HCTR and CIEDs received RM (HCTR-RM). Patients with UC and CIEDs were offered RM optionally (UC-RM). Data from the initial 9 weeks of the study were analysed. Results: Of 850 enrolled patients, 208 were in the HCTR-RM group and 62 in the UC-RM group. The HCTR-RM group was less likely to have alerts of intrathoracic impedance (TI) decrease (*p* < 0.001), atrial fibrillation (AF) occurrence (*p* = 0.031) and lower mean number of alerts per patient associated with TI decrease (*p* < 0.0001) and AF (*p* = 0.019) than the UC-RM group. HCTR significantly decreased the occurrence of alerts in RM of CIEDs, 0.360 (95%CI, 0.189–0.686; *p* = 0.002), in multivariable regression analysis. There were two deaths in the HCTR-RM group (0.96%) and no deaths in the UC-RM group (*p* = 1.0). There were no differences in the number of hospitalised patients between the HCTR-RM and UC-RM group (*p* = 1.0). Conclusions: HCTR significantly reduced the number of patients with RM alerts of CIEDs related to TI decrease and AF occurrence. There were no differences in mortality or hospitalisation rates between HCTR-RM and UC-RM groups.

## 1. Introduction

Heart failure (HF) guidelines recommend regular exercise training in patients with HF to improve symptoms, functional capacity, and to reduce the risk of HF hospitalisation (class 1A recommendation) [1]. Comprehensive cardiac rehabilitation for HF patients includes physical training, patient’s education, health behavior modification and psychosocial support [2,3].

Patients with symptomatic HF (New York Heart Association (NYHA) class II-III) and left ventricular ejection fraction (LVEF) ≤35% despite ≥3 months of optimal medical treatment have indications for an implantable cardioverter–defibrillator (ICD) or cardioverter–defibrillator with cardiac resynchronization therapy (CRT-D) [1]. Individuals with an ICD or CRT-D may have concerns about cardiac rehabilitation due to fear of inappropriate shocks during exercise [4,5]. However, data on different programs of cardiac rehabilitation revealed infrequent appropriate and inappropriate interventions from cardiac implantable electronic devices (CIEDs) in patients during exercise [6,7,8].

In most centers, the majority of the implanted ICDs/CRT-Ds have remote monitoring (RM). According to Heart Rhythm Society (HRS) expert consensus, all patients with CIEDs should be offered RM as an integral part of the standard follow-up management strategy [9].

Studies assessing RM have consistently showed that RM is associated with a significant reduction of the number of appropriate and inappropriate shocks delivered by CIEDs [10]. Furthermore, RM allows earlier detection of actionable events compared with conventional monitoring in patients with CIEDs [11]. It also significantly reduces time to a clinical decision in response to clinical events compared with standard care [12,13]. Of importance, patients with CIEDs who received RM had higher survival rates compared with those who had conventional follow-up [14,15]. Hospitalisations due to worsening HF were also significantly decreased in individuals with RM [15].

Despite clear indications for cardiac rehabilitation in patients with HF and for RM in subjects with CIEDs, data on the influence of cardiac rehabilitation on the number of alerts in RM of CIEDs from multicentre randomised trials are lacking. Therefore, this subanalysis was carried out.

The present subanalysis from the Telerehabilitation in Heart Failure Patients (TELEREH-HF) randomised clinical trial aimed: (i) to compare alerts, including arrhythmic events, recorded in RM in a population of patients with ICDs/CRT-Ds undergoing hybrid comprehensive telerehabilitation (HCTR-RM group) vs. usual care (UC-RM group); and (ii) to compare outcomes of patients in the HCTR-RM and UC-RM groups.

## 2. Materials and Methods

The design and primary results of the TELEREH-HF study (Clinical Trials.gov NCT 02523560) have been published previously [16,17]. Briefly, the TELEREH-HF study was a randomised, prospective, multicentre, open-label, parallel group-controlled trial comparing HCTR and UC in HF patients. The study was conducted in five centres in Poland from June 2015 to June 2017.

Informed consent form was obtained from each patient. The study protocol conforms to the ethical guidelines of the 1975 Declaration of Helsinki.

This study enrolled 850 clinically stable HF patients (NYHA class I-III and LVEF ≤40%) after a hospitalisation due to worsening HF within 6 months prior to randomisation. Eligible individuals were randomised in a 1:1 ratio to either HCTR + UC or to UC only using a web-based randomisation system.

The HCTR intervention encompassed telecare, telerehabilitation and RM of CIEDs. The patients from the HCTR group underwent a 9-week HCTR program (active phase of study). This program included 1 week of telerehabilitation conducted in hospital and an 8-week home-based telerehabilitation programme five times weekly. Each training session included three types of exercises: aerobic endurance training, respiratory muscle training and resistance and strength training. Aerobic endurance training involved: warm-up (from 5 to 10 min), Nordic walking with individually adjusted intensity (gradually prolonged for a maximum of 30–45 min once daily), and cooling down (approximately 5 min). A threshold inspiratory muscle trainer was used during respiratory muscle training (from 5 to 30 min three to five times daily). Resistance and strength training was performed once daily using a TheraBand and lasted up to 15 min. Clinical assessment of patients (questions concerning dyspnoea, fatigue, body weight, blood pressure and taking medicines), evaluation of electrocardiogram (ECG) at rest (EHO mini transmission) and RM of CIEDs (if available) were performed before every session.

Patients in UC were managed according to a routine standard of care during the same period.

HCTR was conducted by a medical team (physicians, physiotherapists, nurses and psychologists). The monitoring system was composed of a remote device for tele-ECG monitoring and supervised exercise training (the telerehabilitation set), a mobile phone and a monitoring centre. The telerehabilitation set included: a special remote ECG transmission device with personalised training sessions (EHO mini device (Pro-Plus Company, Warsaw, Poland)), blood pressure meter and weighting machine. Individual training sessions were programmed in EHO mini devices and included time of exercises, breaks and time for ECG recording (using beeps and luminous signals). At the end of each session, a three-lead ECG at peak exercise was transmitted to the monitoring centre for assessment. Remote programming of new parameters of the training session for the next day was performed after ECG verification and a phone call with the patient in order to assess his/her exercise tolerance.

HCTR group patients with CIEDs (if technical requirements were complied with) received the transmitter—(CardioMessenger (Biotronik, Berlin, Germany); transmitter (Home Monitor) of the CareLink^TM^ network (Medronic, Minneapolis, MN, USA);—Merlin@home^TM^ wireless transmitter, (St. Jude, Little Canada, MN, USA)) which allowed the automatic transmission of data from the implant to a web-based monitoring platform (Biotronik (Berlin, Germany)—Home Monitoring Service Center, Medtronic (Minneapolis, MN, USA)—CareLink-Network, (St. Jude, Little Canada, MN, USA)—Merlin.net^TM^ Patient Care Network). Remote monitoring relied on data acquired automatically on a daily basis by the device, with unscheduled transmission of any predefined alerts to the medical staff in each centre. One physician experienced in RM and CIEDs in each centre was responsible for verification and qualification of arrhythmias based on advanced algorithms for their detection in CIEDs. The physician who assessed arrhythmias in CIEDs was not blinded. According to the study protocol, patients had their alerts in the RM-CIEDs (including arrhythmias) checked before every training session in order to allow them to perform exercises.

Alerts included data on arrhythmias, such as supraventricular tachycardia (SVT), atrial fibrillation (AF), atrial flutter (AFl), ventricular tachycardia (VT), ventricular fibrillation (VF), the occurrence of lung fluid accumulation as indicated by the decrease in the intrathoracic impedance, device integrity (e.g., battery status: end of service (EOS), the elective replacement indicator (ERI), lead impedance, sensing disturbances or pacing/defibrillation threshold out of range), low biventricular pacing (BIV-P) (below 95%) and programming issues (disabling of VT/VF therapy or insufficient capture safety margins).

In this subanalysis, among all eligible patients from the TELEREH-HF study, patients with RM-CIEDs were included. Alerts in RM-CIEDs during the initial 9 weeks of the active phase of the study were analysed. Then, a comparison of the occurrence of alerts between patients from the HCTR-RM group and patients from the UC-RM group was made.

Comparison between the two study groups also included all-cause mortality, and hospitalisations (all-cause, cardiovascular and due to worsening HF). Data from the active phase of the TELEREH-HF study were analysed.

### Statistical Analysis

Results are expressed as means ± standard deviation (SD) or medians and inter-quartile ranges (25–75th) for continuous variables or frequencies and percentages for categorical variables. Comparisons between groups at baseline and during observation (baseline to 9 weeks) were made using a chi-square test of independence or Fisher’s exact test (when the number of expected events was less than five, which occurred for all types of alerts) and Student’s *t*-tests (or Satterthwaite’s method) or Wilcoxon rank-sum tests (skewed distributions), as appropriate. Ordinal variables were compared using Cochran–Mantel–Haenszel modified ridit scores (row mean score statistic). Penalised likelihood logistic regression was used to calculate the adjusted relative risk of rare events (Table 1). The probabilities of alert-free survival were estimated by means of Kaplan–Meier methods. The relationship between the occurrence of the alerts (dependent variable) and potential predictors was assessed using multivariable binary logistic regression analysis (with stepwise method selection and a significance level of 0.05 for entry and 0.05 to stay). All variables listed in Table S3 were included in the multivariable analysis. All statistical tests were performed at α = 0.05 (two-sided). Analyses were performed using SAS software version 9.4.

## 3. Results

### 3.1. Patient Characteristics

From 2015 to 2017, 2333 patients were screened in five centres, and, finally, 425 patients were randomised to the HCTR group and 425 to the UC group. Among 850 enrolled patients, 667 (78.5%) patients had an ICD/CRT-D implanted and 270 of the patients received RM, Supplementary Materials Table S1. Among 425 patients assigned to the HCTR group, 328 (77.2%) patients had an ICD/CRT-D implanted and 213 of them received RM, but for technical reasons, five patients were excluded from the analysis (HCTR-RM group, *n* = 208 (48.9%)). Among 425 patients assigned to the UC group, 339 (79.8%) patients had an ICD/CRT-D and 62 (14.6%) of them had RM (UC-RM group) (Figure S1). Patients from the HCTR group without RM received a CIED without the option of RM or had no Global System for Mobile Communications signal coverage in their homes. Patients from the UC group were offered RM only if it was routine standard of care in a particular centre (in Poland, RM of CIEDs is not reimbursed).

Baseline characteristics of patients with RM in the HCTR group versus the UC group were similar, with the exception of history of myocardial infarction, the use of spironolactone/eplerenone and peak oxygen consumption (Table 2).

### 3.2. Alerts in the Study Groups

One hundred and nineteen RM alerts occurred among 270 patients with CIEDs. Among all of the alerts, 35.3% were AF alerts, 26.1%—VT/VF alerts, 12.6%—low BIV-P (less than 95%) alerts and 4.2%—lead impedance out of range (Figure S2).

Among 208 of the patients in the HCTR-RM group, 36 (17.3%) had alerts, whilst among 62 of the patients in the UC-RM group, 22 (35.5%) had alerts (RR 0.488, 95%CI 0.312–0.764, *p* = 0.002). Patients in the HCTR-RM group were less likely to have thoracic impedance (TI) out of range (RR 0.085, 95%CI 0.018–0.399, *p* < 0.001) and AF alerts (RR 0.335, 95%CI 0.135–0.832, *p* = 0.031) than those in the UC-RM group (Table 1).

A comparison of the RM alert distributions in the study groups showed a smaller number of RM alerts in the HCTR-RM group vs. UC-RM group (*p* = 0.014) (Table 3). When comparing the mean number of RM alerts per patient, patients in the HCTR-RM group were less likely to have alerts associated with thoracic impedance out of range (RR 0.01, 95%CI 0.00–0.02 vs. RR 0.13, 95%CI 0.03–0.23; *p* < 0.0001) and AF occurrence (RR 0.15, 95%CI 0.03–0.27 vs. RR 0.16, 95%CI 0.05–0.28; *p* = 0.019) than those in the UC-RM group (Table 3). Kaplan–Meier curves demonstrated a significantly higher predicted survival without AF alerts in the HCTR-RM group than in the UC-RM group. Evident reduction in AF alerts was observed in the second half of the HCTR period (after about 6 weeks) (Figure S3).

There were no differences in therapies delivered due to alert of VT/VF among two groups (Table S2).

When comparing the patients with AF alerts, three (33%) individuals in the HCTR-RM group had no AF history. All patients in the UC-RM group with AF alerts had previously known AF.

### 3.3. Factors Associated with Occurence of Alerts in RM of CIEDs

There were 58 patients with alerts in RM of CIEDs. Results from univariate and multivariable regression analyses are presented in Table S3 and Table 4. In the univariate regression analysis, factors associated with increased occurrence of alerts in RM of CIED were: non-ischaemic etiology of HF (*p* = 0.045), lower body mass index (*p* = 0.031) and use of digoxin (*p* = 0.020) (Table S3).

In the multivariable regression analysis, digoxin was associated with an increase in the occurrence of alerts in RM of CIEDs (OR 2.398, 95%CI 1.158–4.967; *p* = 0.019). HCTR significantly decreased the occurrence of alerts in RM of CIEDs (OR 0.360, 95%CI 0.189–0.686; *p* = 0.002) (Table 4).

### 3.4. Deaths and Hospitalisations

Among patients in the HCTR-RM group, two individuals died (0.96%), whilst no deaths were observed in the UC-RM group (*p* = 1.0) during the initial 9 weeks of the active phase of the study. One patient died for a non-cardiovascular reason (complications after cholecystectomy). The second death was associated with a cardiovascular reason (stroke).

Sixteen hospitalisations occurred in 10 patients during the active phase of the study. No significant differences occurred in terms of the number of hospitalised patients between the HCTR-RM and UC-RM groups (8; 3.8% vs. 2; 3.2%, respectively, *p* = 1.0). Among all hospitalisations, there were nine cardiovascular hospitalisations (seven in the HCTR-RM group vs. two in the UC-RM group, means per patients on both groups: 0.03, *p* = 0.729), one hospitalisation due to worsening HF in the UC-RM group vs. zero in the HCTR-PM group, and six non-cardiac hospitalisations (five in the HCTR-RM group vs. one in the UC-RM group, mean per patient in both groups: 0.02, *p* = 0.929).

## 4. Discussion

In this subanalysis of the TELEREH-HF study, among patients randomised to the HCTR group, there were significantly fewer alerts associated with decreased TI and AF occurrence when compared with the UC-RM group. Moreover, the HCTR-RM group had a significantly smaller mean number of alerts per patient associated with a decreased TI and AF occurrence.

The most important result of this study is that participation in the HCTR program and less frequent use of digoxin can be recognised as independent factors associated with a reduced frequency of alerts in a group of patients with RM. In one study, digoxin therapy was associated with increased risk of death from any cause in AF patients. It also increases the risk of arrhythmic death, cardiovascular death and stroke. Unfortunately, the mechanisms which are responsible for the association between digoxin therapy and adverse outcomes in AF patients are uncertain [18].

In our study, “lower body mass index (BMI)” was a pro-alert factor only in univariate analysis. The data on the abovementioned relation are scarce. In one study [19], association between the risk of AF and BMI was not linear but U-shaped. “Lower BMI”, in that study, was associated with increased risk of AF. Each 1 kg/m^2^ lower BMI below 20 kg/m^2^ was associated with a 13% higher risk of AF. The annual incidence rate of AF was 3.6 per 1000 person years in the underweight group and was comparable to that of obesity class I (25.0–29.9 kg/m^2^). The increased risk of AF in the underweight group also occurred after adjustment for pre-existing thyroid disease, chronic lung disease and a history of malignancy. According to the available data, there are some biological factors associated with underweight BMI which are involved in the development of AF. The increased level of adiponectin in underweight individuals may be involved in the increased risk of development of AF [20]. The other potential factors which increase the risk of AF seem to be loss of muscle mass and hence loss of myostatin expression [21].

Our observations indicate that HCTR is associated with a significantly lower occurrence of alerts associated with potential fluid overload in the lungs.

Available data on the measurement of TI to predict decompensated HF seem questionable. The earlier reports showed that the monitoring of TI in patients with HF is inversely associated with pulmonary capillary wedge pressure and fluid balance [22]. Automated detection of TI had a sensitivity of over 76% in predicting hospitalisation for fluid overload [22,23]. More recent studies, conducted in a larger number of subjects, reveal that automated detection of TI has lower sensitivity in predicting hospitalisations for decompensation of HF (sensitivity 20.7–41.5%) [24,25]. The overall performance of TI in predicting decompensation of HF is limited due to high inter-patient variability. Moreover, its use should be redirected towards individuals with more advanced symptoms of HF for whom observation of TI has already been shown to be of diagnostic or prognostic value [25]. Interestingly, there is evidence that cardiac rehabilitation is associated with a reduction of the level of B-type natriuretic peptide (BNP), N-terminal pro-B-type natriuretic peptide (NT-pro-BNP) and some new cardiac biomarkers. This association may be due to a decrease in the left ventricular volume overload [26]. Furthermore, cardiac rehabilitation may be associated with improvement in exercise tolerance in patients with HF (reduction in NYHA class, improvement in peak oxygen consumption and an increase in the distance during the six-minute walk test) and improvement in three specific areas of quality of life: physical functioning, role functioning related to physical state and bodily pain [17,27,28,29].

The important factor associated with the reduction of the number of alerts with IT seems to be the design of the TELEREH-HF study. It should be emphasised that HCTR, which was implemented in our study, was innovative and included telecare, telerehabilitation and RM of patients. Patients in the HCTR group were remotely assessed by a physician before exercise training. Their ECG, weight and blood pressure were also evaluated before each telerehabilitation session. This intensified medical care may result in regular optimisation of medical treatment.

In our study, HCTR also significantly reduced the number of patients with RM alerts of CIEDs related to AF occurrence. Among seventeen patients with AF alerts, three had no history of AF. The mean number of alerts per patient was lower in the HCTR-RM group compared to the UC-RM group. The probability of survival without AF alerts was significantly higher in the HCTR-RM group than in the UC-RM group. A more pronounced reduction of AF alerts was observed in the second half of the HCTR duration. A previous study showed that RM of CIEDs allowed early detection and quantification of AF episodes [11,12]. Furthermore, RM of CIEDs had a high sensitivity (>90%) for AF detection [30]. In one prospective, randomised study, cardiac rehabilitation (three sessions per week over 12 weeks) decreased the average AF duration, frequency and severity of symptoms related to AF [31]. In this study, AF was detected in a previously implanted loop recorder in patients without HF. In the routine vs. aggressive risk factor-driven upstream rhythm control for the prevention of early atrial fibrillation in heart failure (RACE 3) study, patients with AF and HF were randomised to comprehensive cardiac rehabilitation. After a 12-month follow-up, sinus rhythm was present significantly more frequently in the intervention group than in the control group (75 vs. 63%) [32]. In the subanalysis of the Heart Failure: A Controlled Trial Investigating Outcomes of Exercise Training (HF-ACTION) study, after a median follow-up of 2.6 years, there were no differences in AF events between the exercise and the control group. However, in this study, only data on AF episodes that were related to hospitalisation or adverse events were collected for analysis [33].

Cardiovascular risk factors (i.e., hypertension, obesity or sleep apnoea) may contribute to the development of AF [34,35]. The CARDIO-FIT study showed that cardiorespiratory fitness predicts the recurrence of arrhythmia in obese patients with symptomatic AF [36]. In this study, patients with significant cardiorespiratory fitness gain demonstrated lower blood pressure, a decrease in mean high-sensitivity C-reactive protein, improved glycemic control, improvement in left ventricle size and diastolic function and a significant reduction in left atrium size. In one study, cardiac rehabilitation reduced the time in AF. It was also associated with a small reduction of body weight [31]. Moreover, there was no reduction of blood pressure and left atrium size. Possibly, the cardiac rehabilitation-related reduction of the time in AF may be associated with different mechanisms than the reduction of modifiable risk factors. Dysfunction of the autonomous nervous system has been implicated in multiple components, leading to pathogenesis of AF [37]. The clinical pattern of vagal-related paroxysmal AF is preferentially observed in the absence of heart disease, whilst sympathetically mediated AF occurs in the presence of any heart disease [38]. Aerobic exercise training, as a non-pharmacological treatment, plays an important role in enhancing sympathovagal balance. In HF patients, exercise training reduces sympathetic activation and thus decreases AF burden [39]. More research is needed to evaluate the effects of comprehensive cardiac rehabilitation in AF patients with HF.

### Limitations

Our study had several limitations. One of them is the small size of the groups. This may be related to the fact that the RM of CIEDs is not reimbursed in Poland, and thus the purchase of RM is covered by the own resources of hospitals. Another limitation is the limited period of time in which RM alerts were collected during 9 weeks of hybrid telerehabilitation. Moreover, the study protocol did not include RM-CIEDs during the follow-up period.

The effects of exercise training tend to be more evident during the prolonged analysis period. In Poland, CIEDs are controlled regularly in the study centres. However, there was no homogeneous protocol for checking them in the study centres after the study was completed. Thus, CIEDs were controlled by the physicians according to local standards. Additionally, patients were implanted with CIEDs from different companies which may have resulted in the lack of homogeneity in the data from RM and differences in the respective algorithms (e.g., TI, detection of AF burden).

## 5. Conclusions

Hybrid comprehensive telerehabilitation significantly reduced the number of patients with RM alerts of CIEDs related to TI decrease and AF occurrence. There were no differences in mortality or hospitalisation rates between the HCTR-RM and UC-RM groups.

## Figures and Tables

**Table 1:** Comparison of the number of patients with alerts in HCTR-RM and UC-RM groups.

Type of Alert	HCTR-RM Group (*n* = 208)	UC-RM Group (*n* = 62)	Relative Risk (95%CI) HCTR-RM vs. UC-RM	*p*	Relative Risk (95%CI) ***	*p* ***
AF, *n* (%)	9 (4.3)	8 (12.9)	0.335 (0.135; 0.832)	0.031 *	0.293 (0.103; 0.829)	0.018
Lead impedance out of range, *n* (%)	2 (1.0)	2 (3.2)	0.298 (0.049; 2.073)	0.227 *	0.346 (0.051; 2.356)	0.209
VT/VF, *n* (%)	13 (6.2)	7 (11.3)	0.554 (0.231; 1.327)	0.179 *	0.535 (0.205; 1.478)	0.204
nsVT, *n* (%)	4 (1.9)	0	NA	0.577 *	2.536 (0.243; 343.770)	0.503
ERI/EOS, *n* (%)	1 (0.5)	1 (1.6)	0.298 (0.019; 4.697)	0.407 *	0.128 (0.005; 2.174)	0.100
TI out of range, *n* (%)	2 (1.0)	7 (11.3)	0.085 (0.018; 0.399)	<0.001 *	0.125 (0.023; 0.489)	0.003
Low BIV-P, *n* (%)	5/77 (6.5)	5/31 (16.1)	0.403 (0.125; 1.294)	0.146 *	0.452 (0.125; 1.628)	0.213
Other, *n* (%)	3 (1.4)	2 (3.2)	0.447 (0.076; 2.616)	0.324 *	0.497 (0.086; 3.216)	0.398
Number of patients with alerts recorded, *n* (%)	36 (17.3%)	22 (35.5%)	0.488 (0.312; 0.764)	0.002 **	0.409 (0.214; 0.786)	0.007

AF, atrial fibrillation; BIV-P, biventricular pacing; CI, confidence interval; ERI/EOS, elective replacement indicator/end of service; HCTR-RM, hybrid comprehensive telerehabilitation group with remote monitoring of cardiac implantable electronic devices; nsVT, non-sustained ventricular tachycardia; TI, thoracic impedance; UC-RM, usual care group with remote monitoring of cardiac implantable electronic devices; VT/VF, ventricular tachycardia/ventricular fibrillation. * Fisher’s exact test, ** chi-square test of independence, *** penalised likelihood logistic regression, adjusted for myocardial infarction and spironolactone/eplerenone and baseline peak VO2.

**Table 2:** Baseline characteristics of patients assigned to the HCTR-RM or UC-RM group.

	HCTR-RM Group (*n* = 208)	UC-RM Group (*n* = 62)	*p*
Gender, male, *n* (%)	189 (90.9)	59 (95.2)	0.278
Age, mean (SD), years	61.3 ± 11	62.2 ± 9	0.563
Left ventricular ejection fraction, mean (SD), %	29.3 ± 6.9	27.9 ± 6.6	0.142
Atrial fibrillation/atrial flutter, *n* (%)	44 (21.1)	15 (24.2)	0.611
Body mass index, mean (SD), kg/m^2^	29.2 ± 4.6	29.2 ± 4.9	0.997
QRS, mean (SD), ms	140.5 ± 34.8	148.2 ± 34.6	0.130
Etiology of heart failure, *n* (%)			
Ischaemic	135 (64.9)	35 (56.4)	0.226
Non-ischaemic	73 (35.1)	27 (43.5)	
Previous medical history, *n* (%)			
Myocardial infarction	126 (60.6)	28 (45.2)	0.031
Percutaneous coronary intervention	100 (48.1)	24 (38.7)	0.194
Coronary artery bypass grafting	34 (16.4)	7 (11.3)	0.330
Valve surgery	11 (5.3)	5 (8.1)	0.376
Hypertension	115 (55.3)	37 (59.7)	0.541
Stroke	15 (7.2)	3 (4.8)	0.772
Diabetes	71 (34.1%)	22 (35.5)	0.844
Chronic kidney disease	34 (16.4)	7 (11.3)	0.330
Hyperlipidaemia	106 (51.0)	24 (38.7)	0.090
Depression, BDI II > 13	40 (23.4)	13 (30.2)	0.353
Functional status, *n* (%)			
NYHA I, *n* (%)	21 (10.1)	7 (11.3)	0.058
NYHA II, *n* (%)	151 (72.6)	36 (58.1)	
NYHA III, *n* (%)	36 (17.3)	19 (30.6)	
NT-pro-BNP, mean (pg/mL)	979.5 (377.7–2235.5)	879.8 (306,3–2325)	0.633
Peak VO2 (mL/kg/min)	16.5 ± 5.1	14.9 ± 4.6	0.024
Treatment, *n* (%)			
Beta blocker	203 (97.6)	61 (98.4)	1.00
ACEI/ARB	199 (95.7)	59 (95.2)	1.00
Digoxin	34 (16.4)	9 (14.5)	0.730
Loop diuretics	163 (78.4)	51 (82.3)	0.507
Spironolactone/eplerenone	178 (85.6)	60 (96.8)	0.017
Aspirin/clopidogrel	111 (53.4)	29 (46.8)	0.362
Anticoagulants	65 (31.2)	24 (38.7)	0.273
NOAC	40 (19.2)	8 (12.9)	0.253
Statins	166 (79.8)	54 (87.1)	0.195
Implantable cardioverter–defibrillator	131 (62.8)	31 (50.0)	0.111
CRT-P	1 (0.5)	1 (1.6)	
CRT-D	76 (36.7)	30 (48.4)	

HCTR-RM, hybrid comprehensive telerehabilitation group with remote monitoring of cardiac implantable electronic devices; UC-RM, usual care group with remote monitoring of cardiac implantable electronic devices; ACEI, angiotensin-converting enzyme inhibitors; ARB, angiotensin receptor blockers; BDI, Beck Depression Inventory; CRT-D, cardiac resynchronization therapy and cardioverter–defibrillator; CRT-P, cardiac resynchronisation therapy; NOAC, non-vitamin K antagonist oral anticoagulants; NYHA, New York Heart Association; NT-pro-BNP, N-terminal prohormone of brain natriuretic peptide; peak VO_2_, peak oxygen consumption; SD, standard deviation.

**Table 3:** Comparison of number of alerts and mean number of alerts per patient in study groups.

	Number of Alerts in:			Mean Number of Alerts per Patient		
	HCTR-RM Group	UC-RM Group	*p*	HCTR-RM Group	UC-RM Group	*p*
Overall alerts	*n* = 73	*n* = 46	-	0.35 (0.20; 0.50)	0.74 (0.40; 1.08)	0.001
AF	32 (43.8)	10 (21.7)	0.014	0.15 (0.03; 0.27)	0.16 (0.05; 0.28)	0.019
Lead impedance out of range	2 (2.7)	3 (6.5)		0.01 (0.00; 0.02)	0.05 (−0.02; 0.12)	0.195
VT/VF	20 (27.4)	11 (23.9)		0.10 (0.02; 0.17)	0.18 (0.00; 0.35)	0.185
nsVT	4 (5.5)	0 (0.0)		0.02 (0.00; 0.04)	0.00	0.274
ERI/EOS	1 (1.4)	1 (2.2)		0.005 (−0.005; 0.014)	0.02 (−0.02; 0.05)	0.366
TI out of range	2 (2.7)	8 (17.4)		0.01 (0.00; 0.02)	0.13 (0.03; 0.23)	<0.0001
Low BIV-P	6 (8.2)	9 (19.6)		0.08 (0.01; 0.16)	0.29 (0.02; 0.56)	0.105
Other	6 (8.2)	4 (8.7)		0.03 (0.00; 0.06)	0.06 (−0.03; 0.16)	0.364

HCTR-RM, hybrid comprehensive telerehabilitation group with remote monitoring of cardiac implantable electronic devices; UC-RM, usual care group with remote monitoring of cardiac implantable electronic devices; BIV-P, biventricular pacing; AF, atrial fibrillation; VT/VF, ventricular tachycardia/ventricular fibrillation; nsVT, non-sustained ventricular tachycardia; ERI/EOS, elective replacement indicator/end of service; TI, thoracic impedance.

**Table 4:** Independent predictors of occurrence of alerts in RM of CIEDs in all patients with RM.

Step:	Variable	OR (95%CI)	*p*
1	Group (HCTR-RM vs. UC-RM)	0.360 (0.189–0.686)	0.002
2	Digoxin	2.398 (1.158–4.967)	0.019

AUC = 0.641, LR < 0.001, residual chi^2^, *p* = 0.684. HCTR-RM—hybrid comprehensive telerehabilitation group with remote monitoring of cardiac implantable electronic implantable devices; UC-RM, usual care group with remote monitoring of cardiac implantable electronic implantable devices; RM, remote monitoring; CIEDs, cardiac implantable electronic devices.

## Supplementary Materials

The following are available online at https://www.mdpi.com/2077-0383/9/11/3729/s1, Table S1: Baseline characteristics of patients with RM and without RM in the TELEREH-HF study; Table S2: Number of patients with delivered CIED therapy in study groups; Table S3: Factors associated with occurrence of alerts in RM of CIEDs (univariate analysis); Figure S1: Subanalysis of TELEREH-HF study flow diagram; Figure S2: Alerts in patients with cardiac implantable electronic devices and remote monitoring; Figure S3: Probability of survival without atrial fibrillation alerts.
