# Supplementary material for: Remote Monitoring of Cardiac Implantable Electronic Devices in Patients Undergoing Hybrid Comprehensive Telerehabilitation in Comparison to the Usual Care. Subanalysis from Telerehabilitation in Heart Failure Patients (TELEREH-HF) Randomised Clinical Trial
Source: J Clin Med. 2020 Nov 20;9(11):3729. doi: 10.3390/jcm9113729 (PMC7699808; doi:10.3390/jcm9113729)
Supplement: Supplementary file 1 [file jcm-09-03729-s001.pdf]

Supplementary materials

Table 1. Baseline characteristics of patients with RM and without RM in TELEREH-HF study.

	With RM Group (*n* = 270)	Without RM Group (*n* = 580)	*p*
**Gender, male, *n* (%)**	248 (91.8)	505 (87.1)	**0.041**
**Age, mean (SD), years**	61.5 ± 10.4	62.8 ± 10.6	0.089
**Left ventricular ejection fraction, mean (SD), %**	29.0 ± 6.9	31.4 ± 7.0	**<0.001**
**Atrial fibrillation/atrial flutter, *n* (%)**	59 (21.8)	100 (17.2)	0.108
**Body Mass Index, mean (SD), kg/m^2**	29.0 ± 5.0	28.9 ± 4.8	0.620
**QRS, mean (SD), ms**	142.3±34.8	131.8±34.7	**<0.001**
**Etiology of heart failure, *n* (%)**			
• **Ischaemic**	170 (63.0)	385 (66.4)	0.330
• **Non-ischaemic**	100 (37.0)	195 (33.6)	
**Previous medical history, *n* (%)**			
**Myocardial infarction**	154 (57.0)	339 (58.4)	0.698
**Percutaneous coronary intervention**	124 (45.9)	272 (46.9)	0.792
**Coronary artery bypass grafting**	41 (15.2)	99 (17.1)	0.491
**Valve surgery**	16 (5.9)	48 (8.3)	0.227
**Hypertension**	152 (56.3)	382 (65.9)	**0.007**
**Stroke**	18 (6.7)	43 (7.4)	0.694
**Diabetes**	93 (34.4%)	198 (34.1)	0.930
**Chronic kidney disease**	41 (15.2)	108 (18.6)	0.220
**Hyperlipidaemia**	130 (48.2)	266 (45.9)	0.534
**Depression, BDI II > 13**	53 (24.8)	132 (26.4)	0.658
**Functional status, *n* (%)**			
**NYHA I *n* (%)**	28 (10.4)	76 (13.1)	0.527
**NYHA II *n* (%)**	187 (69.3)	390 (67.2)	
**NYHA III *n* (%)**	55 (20.4)	114 (19.7)	
**NT pro BNP, mean (pg/mL)**	970 (371 - 2264)	871 (386 – 1936)	0.499
**Peak VO2 (mL/kg/min)**	16.1 ± 5.0	17.1 ± 6.4	**0.021**
**Treatment, *n* (%)**			
**Beta-blocker**	264 (97.8)	561 (96.7)	0.397
**ACEI/ARB**	258 (95.6)	535 (92.2)	0.072
**Digoxin**	43 (15.9)	61 (10.5)	**0.025**
**Loop diuretics**	214 (79.3)	436 (75.2)	0.191
**Spironolactone/eplerenone**	238 (88.1)	461 (79.5)	**0.002**
**Aspirin/clopidogrel**	140 (51.8)	345 (59.5)	**0.036**
**Anticoagulants**	89 (33.0)	164 (28.3)	0.164
**NOAC**	48 (17.8)	85 (14.7)	0.243
**Statins**	220 (81.5)	476 (82.1)	0.836
**Patients with CIED**	270 (100.0)	406 (70.0)	**<0.001**
**Implantable cardioverter-defibrillator**	162 (60.0)	270 (66.5)	0.113
**CRT-P**	2 (0.7)	6 (1.5)	
**CRT-D**	106 (39.3)	130 (32.0)	

RM, remote monitoring of cardiac implantable electronic devices; ACEI, angiotensin converting enzyme inhibitors; ARB, angiotensin receptor blockers; BDI, Beck Depression Inventory; CRT-D, cardiac resynchronization therapy and cardioverter-defibrillator; CRT-P, cardiac resynchronization therapy; NOAC, non vitamin K antagonist oral anticoagulants; NYHA, New York Heart Association; NT pro BNP, N-terminal prohormone of brain natriuretic peptide; peak VO_2, peak oxygen consumption; SD - standard deviation.

Table S2. Number of patients with delivered CIEDs therapy in study groups.

	HCTR-RM *n* = 208	UC-RM *n* = 62	P
**Number of patients with VT/VF alerts, *n* (%)**	13 (6.2)	7 (11.3)	
**Type of intervention :**			
**Without**	9 (69.2%)	3 (42.8%)	0.474
**ATP**	3 (23.1%)	2 (28.6%)	
**CV**	1 (7.7%)	1 (14.3)	
**ATP+CV**	0	1 (14.3)	

HCTR-RM, hybrid comprehensive telerehabilitation group with remote monitoring of cardiac implantable electronic devices; UC-RM, usual care group with remote monitoring of cardiac implantable electronic devices; CIEDs, cardiac implantable electronic devices; VT/VF, ventricular tachycardia/ventricular fibrillation; CV,cardioversion; ATP, antitachycardia pacing.

Table S3. Factors associated with occurence of alerts in RM of CIEDs (univariate analysis).

	Patients without Alerts (*n* = 212)	Patients with Alerts (*n* = 58)	P
**Gender, male, *n* (%)**	195 (92.0)	53 (91.4)	0.793
**Age, mean (SD), yrs**	61.3 ± 10.4	62.2 ± 10.7	0.564
**Left ventricular ejection fraction, mean (SD), %**	29.3 ± 6.8	27.9 ± 7.0	0.164
**Atrial fibrillation or atrial flutter, *n* (%)**	41 (19.3)	18 (31.0)	0.056
**Body Mass Index, mean (SD), kg/m^2**	29.4 ± 5.3	27.8 ± 4.1	**0.018**
**QRS, mean, ms**	140.2 ± 34.9	150.1 ± 33.9	0.056
**Peak VO2, mean, ml/kg/min**	16.3 ± 5.3	15.5 ± 4.1	0.186
**NT proBNP, mean, pg/mL**	942 (361 – 2027)	1257 (434 -2379)	0.301
**Etiology of heart failure, *n* (%)**			
• **Ischaemic**	140 (66.0)	30 (51.7)	**0.045**
• **Non-ischaemic**	72 (34.0)	28 (48.3)	
**Past medical history, *n* (%)**			
**Myocardial infarction**	126 (59.4)	28 (48.3)	0.128
**Percutaneous coronary intervention**	99 (46.7)	25 (43.1)	0.626
**Coronary artery bypass grafting**	33 (15.6)	8 (13.8)	0.739
**Valve surgery**	10 (4.7)	6 (10.3)	0.120
**Hypertension**	122 (57.5)	30 (51.7)	0.428
**Stroke**	11 (5.2)	7 (12.1)	0.076
**Diabetes**	76 (35.8%)	17 (29.3)	0.353
**Chronic kidney disease**	34 (16.0)	7 (12.1)	0.455
**Hyperlipidaemia**	103 (48.6)	27 (46.5)	0.784
**Functional status, *n* (%)**			
**NYHA I**	22 (10.4)	6 (10.3)	0.998
**NYHA II**	147 (69.3)	40 (69.0)	
**NYHA III**	43 (20.3)	12 (20.7)	
**NYHA III vs NYHA II /NYHA I**			
**Treatment, *n* (%)**			
**Beta-blocker**	207 (97.6)	57 (98.3)	1.00
**ACEI/ARB**	204 (96.2)	54 (93.1)	0.294
**Digoxin**	28 (13.2)	15 (25.9)	**0.020**
**Loop diuretics**	168 (79.2)	46 (79.3)	0.991
**Spironolactone/eplerenone**	187 (88.2)	51 (87.9)	0.954
**Aspirin/clopidogrel**	111 (52.4)	29 (50.0)	0.750
**Anticoagulants**	68 (32.1)	21 (36.2)	0.553
**NOAC**	37 (17.4)	11 (19.0)	0.789
**Statins**	173 (81.6)	47 (81.0)	0.921

ACEI, angiotensin converting enzyme inhibitors; ARB, angiotensin receptor blockers; BDI, Beck Depression Inventory; CRT-D, cardiac resynchronization therapy and cardioverter-defibrillator; CRT-P, cardiac resynchronization therapy; HCTR-RM, hybrid comprehensive telerehabilitation group with remote monitoring of CIEDs; NOAC, non vitamin K antagonist oral anticoagulants; NYHA, New York Heart Association class; peak VO_2, peak oxygen consumption; SD, standard deviation; UC-RM, usual care group with remote monitoring of CIEDs; RM, remote monitoring; CIEDs, cardiac implantable electronic devices.

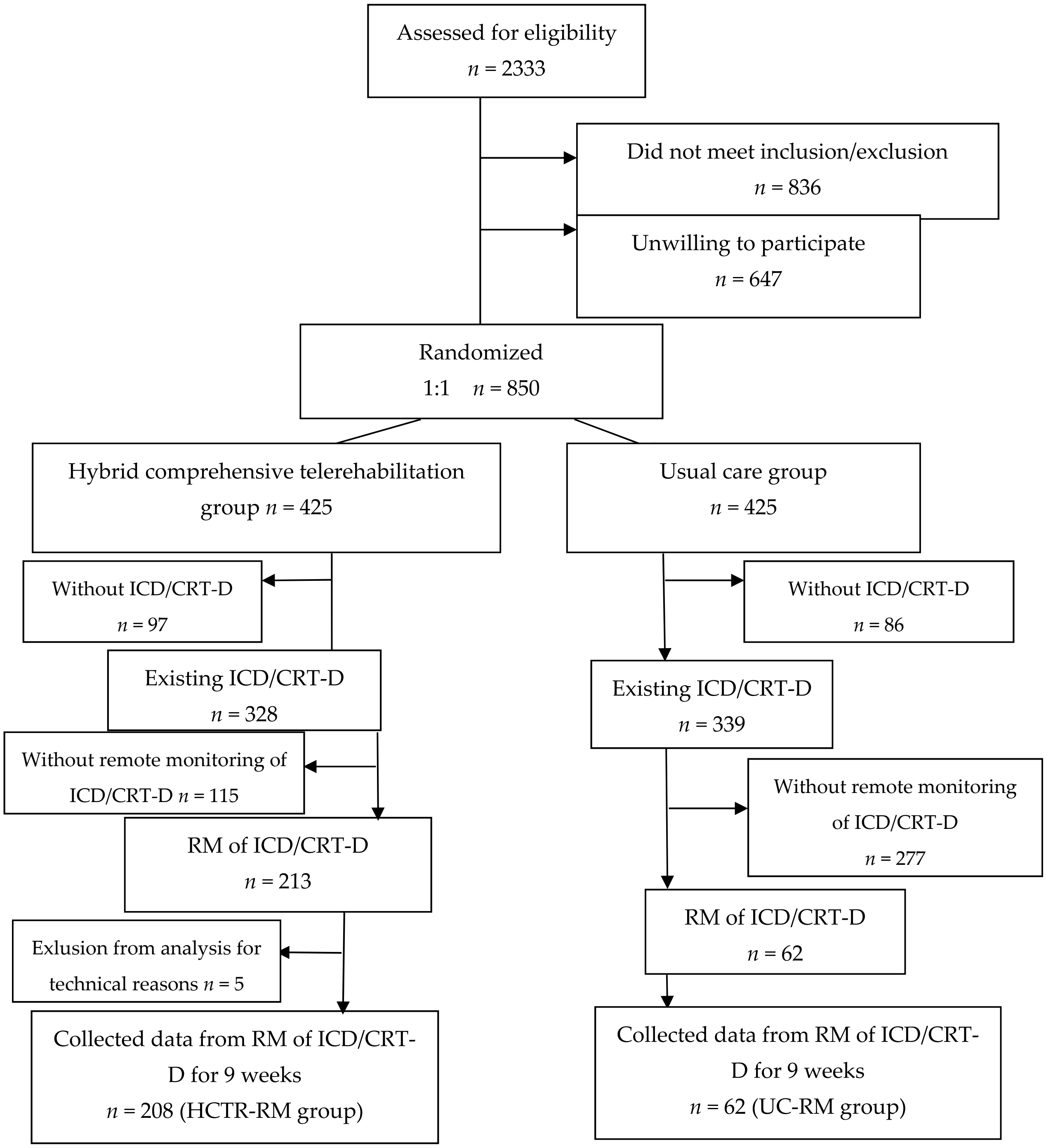

Figure S1. Subanalysis of TELEREH-HF study flow diagram. HCTR-RM, hybrid comprehensive telerehabilitation group with remote monitoring of CIEDs; UC-RM, usual care group with remote monitoring of CIEDs; ICD, cardioverter-defibrillator; CRT-D, cardiac resynchronization therapy and cardioverter-defibrillator.

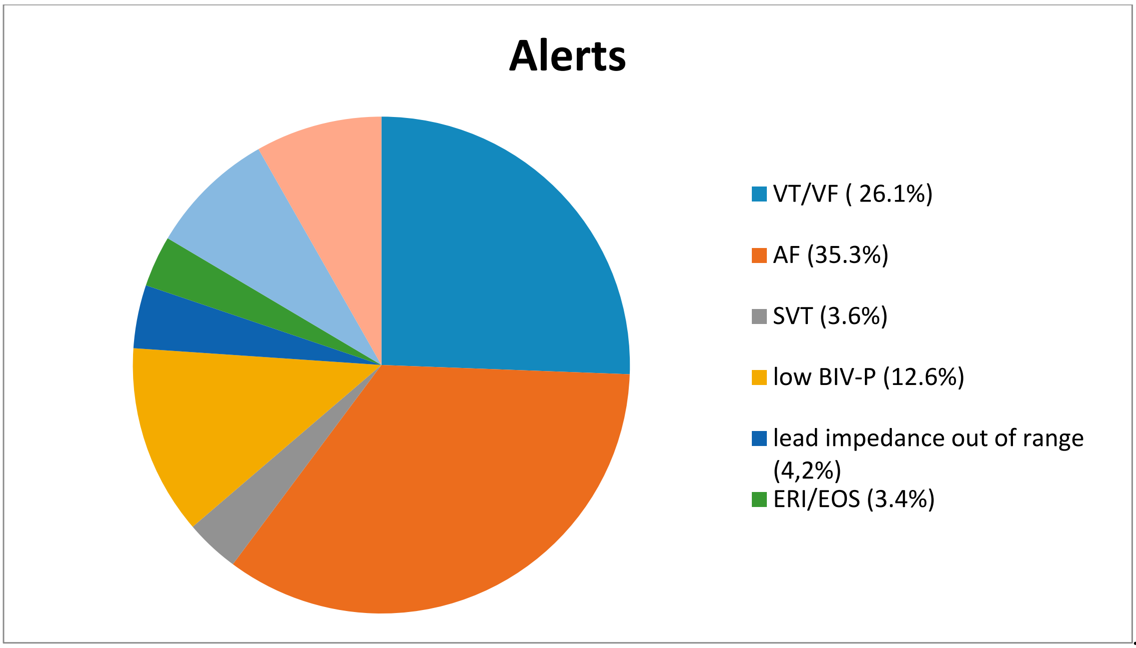

Figure S2. Alerts in patients with Cardiac Implantable Electronic Devices and Remote Monitoring (*n* = 119). AF, atrial fibrillation; BIV-P, percentage of biventricular pacing; CIED - EOS/ERI - end of service/ elective replacement indicated; SVT, supraventricular tachycardia; TI, thoracic impedance out of range; VT/VF, ventricular tachycardia/ ventricular fibrillation.

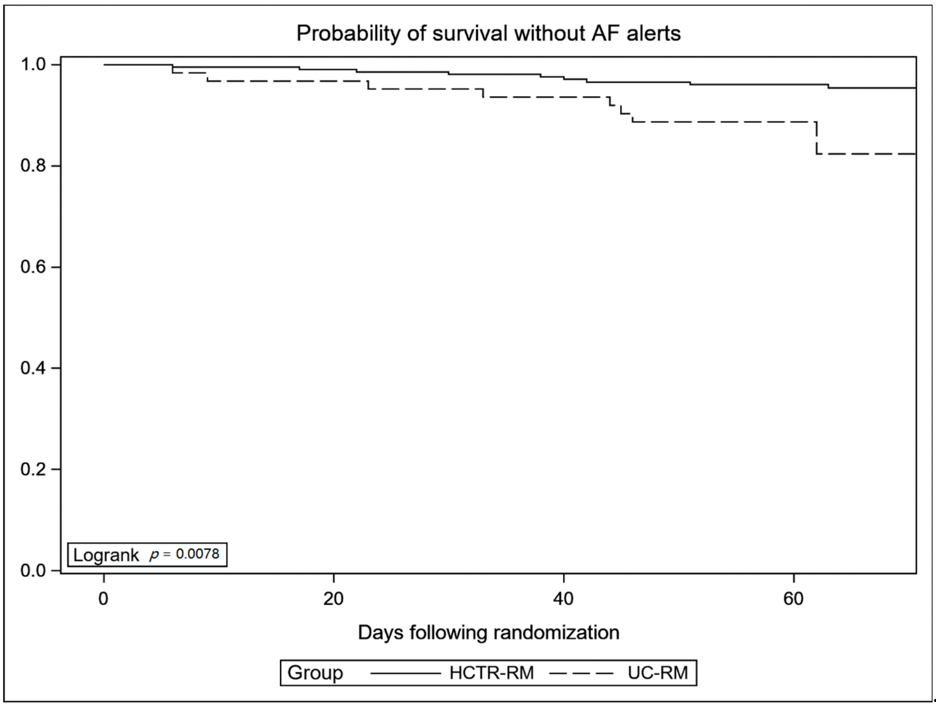

Figure S3. Probability of survival without atrial fibrillation alerts. HCTR-RM, hybrid comprehensive telerehabilitation group with remote monitoring of CIEDs; UC-RM, usual care group with remote monitoring of cardiac implantable electronic devices; AF, atrial fibrillation.
